# SPHERpower: MSC spheroid-based bioequivalent lead to the efficient restoration of the scarred vocal folds

**DOI:** 10.1186/s13287-026-05067-5

**Published:** 2026-05-30

**Authors:** Anastasia Shpichka, Mikhail Svistushkin, Yana Khristidis, Polina Bikmulina, Natalia Serejnikova, Alexey Fayzullin, Gaia Lebedeva, Alesia Bakulina, Anna Zolotova, Igor Zinchenko, Nastasia Kosheleva, Nafisa Fayzullina, Mikhail Belovolov, Yuri Efremov, Anna Solovieva, Valeriy Svistushkin, Peter Timashev

**Affiliations:** 1 Institute for Regenerative Medicine, Sechenov University, Moscow, Russia; 2 Department for ENT Diseases, Sechenov University, Moscow, Russia; 3 Fiber Optics Research Ctr., Moscow, Russia; 4 Department of Polymers and Composites, N.N. Semenov Federal Research Center for Chemical Physics, Russian Academy of Sciences, Moscow, Russia; 5 Department of Biological Chemistry, Sechenov University, Moscow, Russia

**Keywords:** Vocal folds, Mesenchymal stromal cells, Spheroid, PEG-fibrin, Bioequivalent, Regenerative medicine, Tissue engineering

## Abstract

Cell-based therapy has become an attractive option to restore the vocal folds (VF) after scarring. Despite that first clinical trials using mesenchymal stromal cells (MSC) suspension has been launched, there is still an issue of retaining cells at implantation site to prolong their effects. Therefore, cell spheroids are considered to be a part of next generation bioequivalents. This study aims to reveal the safety and efficacy of MSC spheroid-based bioequivalent in treating VF scars in comparison with cell suspension. To evaluate this approach in vivo, a rabbit vocal fold scar model was established in 48 male rabbits, which were allocated to four groups: untreated control, PEG-fibrin, PEG-fibrin with MSC suspension, and PEG-fibrin with MSC spheroids. In 3 months post operation, in group where the MSC suspension-based bioequivalent was implanted, the mature connective tissue was located beneath the epithelium without dystrophic changes and had a relatively loose ECM; the thickness of the lamia propria was higher than that in the intact VF. In group where we used the MSC spheroid-based bioequivalent the loose connective tissue consisted of longitudinally oriented collagen fibers, spindle-shaped fibroblasts; the lamia propria was relatively thin (116.42 ± 42.77 μm) and loose and did not differ from the intact VF. Both types enabled the VF restoration; nevertheless, the use of MSC spheroids lead to more efficient regeneration: the tissue architectonics and mechanical properties of the treated VF was more similar to those of the intact VF.

**Supplementary Information:**

The online version contains supplementary material available at 10.1186/s13287-026-05067-5.

## Introduction

Nowadays, treating scar lesions of the vocal folds (VF) using cell-based products has become an approach of high interest among ENT specialists. Compared to traditional methods, their main effects are based on modulating regenerative processes in a damaged area that ensures the inhibition of fibrosis, the decrease in the level of inflammation and scar area, the restoration of extracellular matrix (ECM) and, consequently, tissues’ vibration properties [51]. The results of the first clinical trials using products based on mesenchymal stromal cells (MSC) have already demonstrated their safety and high efficacy [20, 35].

As the mechanism of their action is mainly paracrine, one of the therapy goals is to enable cell retaining for more than several days; so, the cell secretome could orchestrate the tissue regeneration longer and ensure the better anatomical and functional restoration. To achieve this, one of the common strategies is the use of various carrier materials (e.g. fibrin, hyaluronic acid, collagen, etc.) [10, 22, 24, 27], and the first successful results were even reported more than 10 years ago [34, 41]. Particularly, we also previously showed that the bioequivalent fabricated using PEG-fibrin conjugates and human bone marrow-derived MSC possessed better outcomes then the single cell suspension [42, 43, 49]. 

Nevertheless, in some cases, adding a carrier may be insufficient, and the patient have to undergo re-implantation. Therefore, there is an increasing interest in using cell spheroids (also called micro-tissues) – a 3D spherical structure formed by cells with tight intercellular junctions and ECM. Compared to 2D cultures, the advantages of using spheroids are not limited to their form. Spheroids maintain stemness and a high differentiation potential of MSCs [23, 33, 46, 48]. Several studies showed that the secretome of MSC spheroids contains higher concentration of vascular endothelial growth factor A (VEGFA), interleukin-6 and 8 (IL-6, 8), chemokine (CXC motif) ligand 1 (CXCL1) than that of monolayers [14]. Their exosomes up-regulated SOX2 and NANOG mRNAs enabling stemness maintenance and miR126 and miR146a – angiogenesis [52]. Moreover, the bioequivalent consisting of a carrier biomaterial and MSC spheroids was proven to possess more pronounced effects, particularly promoting muscle [16, 17] and cartilage [53] tissue regeneration, wound healing [37, 40], neurogenesis [19]. Thus, this study aims to demonstrate the safety and efficacy of such bioequivalent in treating VF scar lesions.

## Materials and methods

### Cell culture

We used human mesenchymal stromal cells (MSC) derived from the alveolar mucosa of the gum. The material was provided from patients after signing an informed consent by the Biobank at the Sechenov University (Moscow, Russia). The cells were isolated in accordance with a standard procedure as described elsewhere [7 and 12]. We cultivated cells using a DMEM/F12 (1:1) medium containing glutamine (2 mM L, Sigma Aldrich, Germany), gentamicin (50 µg mL^− 1^, PanEco, Russia), insulin-transferrin-selenite (1:100, Prospec, Israel), fibroblast growth factor-2 (20ng/mL, Sci-Store, Russia), and fetal bovine serum (FBS) (10%, HyClone, USA) under standard conditions (37 °C; 5% CO2). The medium was changed every 2–3 days; and only cells younger than Passage 4 or equal were included in experiments carried out. The MSC phenotype was proven by the analysis of surface markers and ability to differentiate into the three lineages as described previously [12].

### Spheroids’ formation and characterization

Cell spheroids were fabricated using non-adhesive agarose plates replicated from 3D Petri Dish molds (Microtissues, USA). The cell suspension was added to the plate (2 × 10^3^ cells per well) and then cultured using the medium described above for 3 days under standard conditions (37 °C; 5% CO2). The cells were monitored using a light phase-contrast microscope AxioVert.A1 (Carl Zeiss, Germany).

To reveal the viability of cells within a spheroid, samples were stained using Calcein-AM and propidium iodide (Live/Dead Assay kit, Sigma Aldrich, Germany) in accordance with manufacturer’s instructions. Nuclei were additionally treated with Hoechst 33,258 (Sigma Aldrich, USA) for 15 min. Images were obtained using a laser scanning confocal microscope LSM 880 equipped with an AiryScan module and a GaAsP detector (Carl Zeiss, Germany).

For immunocytochemical staining, the samples were fixed in 4% paraformaldehyde solution (Sigma-Aldrich, Germany) overnight at 4 °C. After triple washing with PBS, they were permeabilized using 0.2% Triton X-100 solution (PanReac AppliChem, USA) for 10 min and blocked with 5% FBS solution for 10 min. Then primary antibodies against vimentin (Abcam, UK) were added, and the samples were placed at room temperature for 4 h and incubated with secondary antibodies conjugated with Alexa Fluor 488 (ThermoFisher Scientific, USA) after washing. Actin was stained using phalloidin conjugated with Alexa Fluor 594 (Invitrogen, USA), cell nuclei – as described above. Then the samples were analyzed using a laser scanning confocal microscope LSM 880 (Zeiss, Germany).

For scanning electron microscopy (SEM), the samples were fixed in 3% glutaraldehyde overnight at + 4 °C and washed three times with PBS. Then they were treated with 1% osmium tetroxide solution at room temperature for 1 h, dehydrated using ethanol range (50%, 70%, 80%, and 96% – twice for 5 min each) and acetone, and dried using a critical point drier. Images was taken using a scanning electron microscope CamScan-S2 (Cambridge Instruments, UK).

For transmission electron microscopy, the samples were embedded within resin as blocks and sliced using a ultramicrotome LKB V (LKB Produkter, Sweden). Ultrathin sections were treated with 1% uranyl acetate solution at room temperature for 1 h and studied using a transmission electron microscope JEM-1011 (JEOL, Japan).

### Hydrogel system preparation and equivalent fabrication

A hydrogel system used is based on PEG-fibrin conjugates and was prepared in accordance with previously described procedure [1, 6, 47]. An equivalent was formed prior to the surgery while spheroids mixed with the prepared fibrinogen solution and then added thrombin, and kept in sterile 1.5 mL Eppendorf tubes. The cell viability was assessed by Live/Dead staining (Sigma Aldrich, Germany), AlamarBlue (Invitrogen, USA), and PicoGreen (Invitrogen, USA) assays as described previously [6, 12].

### In vivo experiments

**Fig. 1:**
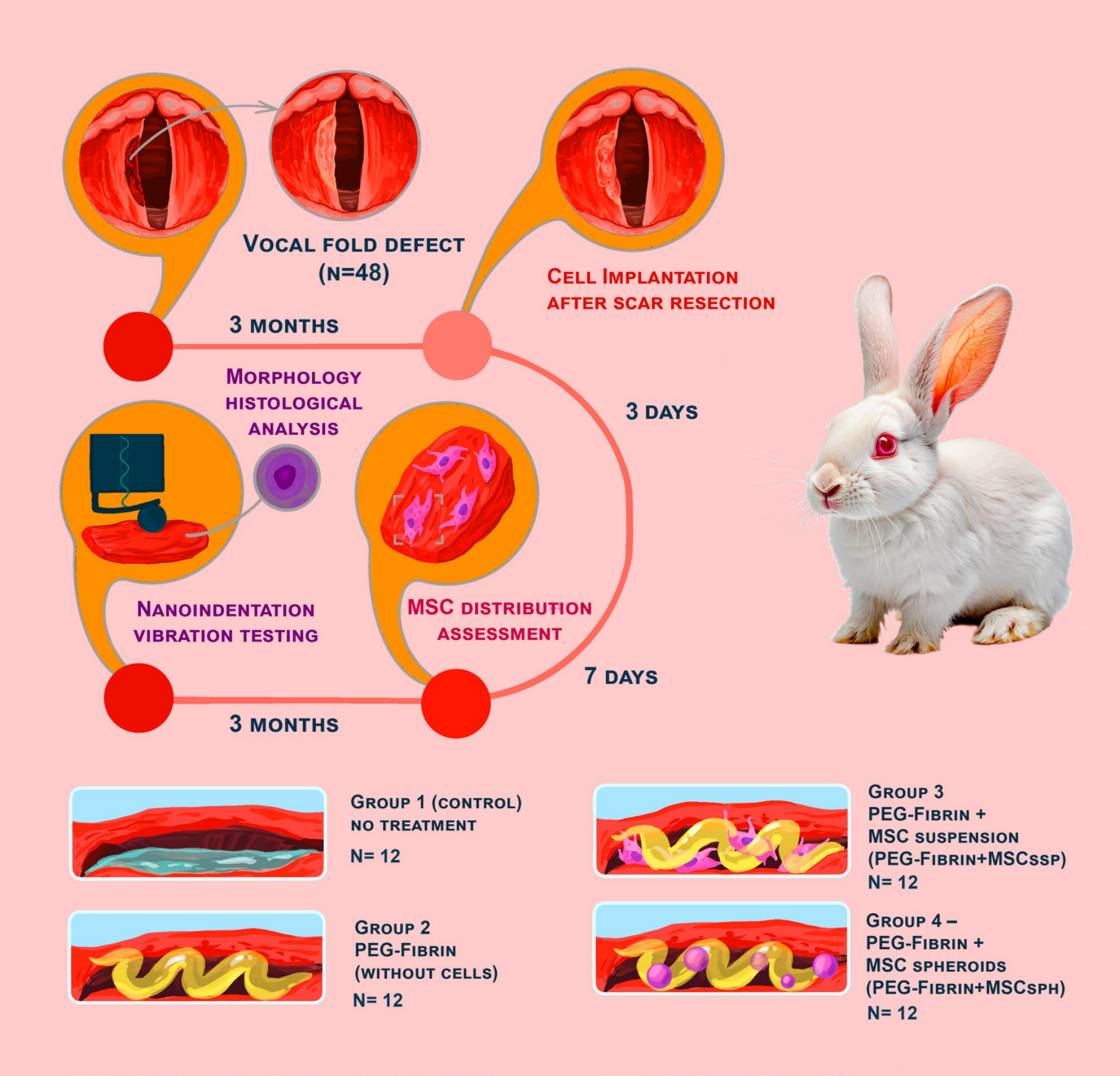
Experiment design. The bioequivalent proposed consists of spheroids from human MSC derived from the alveolar mucosa of the gum and PEG-fibrin-based hydrogel system. The samples were implanted orthotopically into a secondary VF wound created after scar excision in rabbits. A VF scar was first surgically induced and allowed to mature for 3 months. The scar tissue was then excised to generate a secondary defect, followed by implantation of the corresponding samples. The outcomes were evaluated in 3 and 7 days and 3 months after implantation (histological estimation and mechanical measurements)

The animal experiments were divided into two blocks (hetero- and orthotopic implantation) (Fig. 1) and described below in accordance with ARRIVE guidelines. The sample size and relevant methods to assess effects were chosen based on our previous experience and literature analysis [49, 51]. Due to the homogeneity of the animal’s population, we did not used inclusion and exclusion criteria. The animal randomization to form groups was performed using a random number generator. To minimize bias and confounders, we blinded all materials and relevant items at every stage. A group name was created as a code, and an animal’s one – group code and number in an ascending order. While implanting and measuring, we altered samples and animals.

The subcutaneous implantation was performed to male Wistar rats (3-month-old, 300–400 g) (*n* = 24: 12 in Group 1 (PEG-Fibrin) and 12 in Group 2 (PEG-Fibrin+MSCsph; 1 × 10^3^ spheroids/ml (2 × 10^3^ cells per spheroid)). The analgosedation was performed by an intravenous injection of tiletamine-zolazepam and an intramuscular injection of xylazine solution based on 10–15 mg/kg and 1–2 mg/kg, respectively. The sample (500 µl) was administered subcutaneously using a 23G needle. No additional therapy was carried out in the post-surgical period. 7 and 21 days after the injection, 6 animals from each group were removed from the experiment by means of excessive intramuscular and intravenous administration of xylazine (0.5 mL, 1–2 mg/kg) and tiletamine-zolazepam (3 mL, 50 mg/mL + 50 mg/mL) solution, respectively. The tissues surrounding the implant were visually assessed, and explants were taken.

Then we used a VF scar model in rabbits (males, “Sovetskaya Shinshila”, 8-month-old, 2500–4000 g, *n* = 48). The experiment was carried out in two consecutive stages. At the first stage, a unilateral injury of the left VF was surgically created to establish a model of chronic VF scarring. Three months later, during the second stage, the scarred area was identified, excised to create a secondary defect, and subsequently treated with the corresponding implant. The animals were allocated to four experimental groups: Group 1 (control, *n* = 12) – no treatment; Group 2 (*n* = 12) – PEG-Fibrin (without cells), Group 3 (*n* = 12) – PEG-Fibrin + MSC suspension (PEG-Fibrin+MSCssp), Group 4 (*n* = 12) – PEG-Fibrin + MSC spheroids (PEG-Fibrin+MSCsph) (Fig. 1). Additionally, six intact VFs was analyzed.

All surgical manipulations were performed under aseptic conditions in an operating room at the vivarium (Sechenov University, Moscow, Russia) as described previously [49]. To ensure analgesia, sedation, and muscle relaxation, analgosedation was induced by intramuscular administration of tiletamine-zolazepam (10–15 mg/kg) and xylazine (1–2 mg/kg). Direct laryngoscopy was performed using a Miller C size 1 neonatal blade and a Karl Storz-Hopkins 0°, 4.0 mm rigid endoscope. At the first stage, a unilateral defect was created in the left VF by excising the mucosa and lamina propria over approximately one-third of the VF length at a distance of 3–4 mm from the anterior commissure using cup forceps. When necessary, blood clots were removed and hemostasis was achieved with a cotton swab soaked in adrenaline solution. In the postoperative period, cefotaxime was administered intramuscularly at 75 mg/kg once daily for 5 days. Three months after the second procedure was performed. The scar borders were determined by visual and tactile assessment, after which the scar tissue was excised using cup forceps. Immediately thereafter, the corresponding material (500 µl) was injected into the margins of the secondary wound and the lamina propria using a needle for endoscopic rhino-sinus surgery. The correct injection depth and site were assessed by the formation of a characteristic infiltrate corresponding to the injected volume.

The bioequivalents were prepared in the operating room directly before the surgery by mixing equal volumes of their components (thrombin and PEG-fibrinogen without and with MSC (2 × 10^6^ cells/ml) or cell spheroids (1 × 10^3^ spheroids/ml (2 × 10^3^ cells per spheroid) in a 1.0 mL syringe (based on our previous experience [49]. All animals survived and were humanely euthanized on day 3, 7, and 90: rats – using the CO2 Euthanizer (AWTech, Russia); rabbits were sedated by Zoletil 100 (Vibrac, Franc; 10 mg/kg intravenously), and then their respiratory centers were inhibited by lidocaine hydrochloride (10%; 100 mg/kg; Organika, Russia). We excised the laryngeal-tracheal complex from the adjacent tissues using a surgical kit and then isolated the VF by incising above and below them.

#### Mechanical properties and vibration activity

The distribution of local mechanical VF characteristics was assessed using a MicroTester MT G2 (CellScale, Canada). The dissected samples were placed into PBS, immobilized on glass slides, and measured thrice at an indentation mode (800-µm depth) using a spherical microindenter with a diameter of 1.5 mm attached to the end of a 48-mm long tungsten beam. The Young’s moduli in the experimental groups were compared with that of the intact tissue. The Young’s modulus was calculated according to the Hertz’s model using the Matlab software. The obtained force-displacement curves were processed as follows to extract Young’s moduli (mean ± standard deviation (SD), kPa):

$$\:F=\frac{4}{3}\frac{E}{1-{v}^{2}}{\delta\:}^{\frac{3}{2}}\sqrt{R}$$

E – Young’s modulus, ν –Poisson’s ratio (it was taken equal to 0.5), R - indenter radius.

The VF vibrations were measured using a non-contact set-up previously developed and described in [4]. It includes the following parts: a silicone cone holder, a fiber optic probe, a microphone, an optoelectronic laser unit with an auxiliary red laser, a two-channel sound card with analog-to digital converter, and a laptop with customed software. The samples were fixed in the silicone cone holder with a flat top. The fiber probe was placed at a 1-mm distance from their mid-surface using mechanical micromanipulators and enabled to record only the VF vibrations (not their sound). A low‑Q Fabry–Pérot interferometer acts as the sensitive element; it results from the combination of the fiber tip’s flat end (with its 4% Fresnel reflection) and the initial boundary of the vibrating VF surface, which is coated with mucus. Using a three‑coordinate xyz stage, we mechanically moved the sample to vary the position of the optical sensing point. The laser radiation (1.55 μm, 100 µW) is safe and possess no thermal effect on tissues. The amplitudes, frequencies (f1, f2, f3, Hz), and shapes of the excited microoscillations of the closed VF in the experimental groups were measured at least three times and compared with those of the native tissue. The closure was not absolute: the VF were able vibrate freely under passing air. After being measured, we dissected the samples and dipped into a 10% neutral buffered formalin solution for 24 h.

The statistical analysis was carried out using GraphPad Prism software version 8.0.1 for Windows (GraphPad Software, Inc.). The normality of data distribution was assessed using the Shapiro-Wilk test, and the differences among the groups were calculated using the ANOVA dispersion analysis and the Tukey test (*p* < 0.05).

#### Histological analysis

The fixed samples after heterotopic implantation (rats, *n* = 6 in each group for each time point, total number of samples was 24) and after orthotopic implantation (rabbits, 4 VFs for each time point of each study group and 6 intact VFs, total number of VFs was 48 injured VF and 6 intact ones (54)) were immobilized in paraffin blocks and sliced into 4-µm-thick sections stained with hematoxylin and eosin (H&E), orcein (elastic fibers), Mallory’s trichrome stain and Picrosirius red (PSR) (collagen fibers). They were further analyzed via standard and polarized light microscopy using a microscope DM4000 B LED equipped with a digital camera DFC7000 T (Leica Microsystems, Germany). Histological samples were digitized using a Hamamatsu NanoZoomer S20 slide scanner (Hamamatsu, Japan) at 400× magnification. Digital slide images were analyzed using the NDP.view 2 software (Hamamatsu, Japan).

For immunohistochemical study, the sections from the same paraffin blocks (*n* = 54) were deparaffinized, incubated with 3% H2O2 for 10 min, heated in a sodium citrate buffer for 30 at 80 °C, and blocked with Background Block (Cell Marque, USA). Then, the samples from days 3 and 7 after the implantation (4 VFs for each study group) were incubated with monoclonal mouse antibodies to antinuclear human antigen (Anti-Nuclei Antibody, clone 235-1). All samples (4 VFs for each time point of each study group) were incubated with mouse monoclonal primary antibodies to α-smooth muscle actin (α-SMA) (A2547, Merck, USA). The detection was carried out using polyclonal HRP-conjugated (Invitrogen, USA), biotin-conjugated (Invitrogen, USA) secondary goat antibodies and streptavidin-HRP peroxidase conjugate (Thermo Fisher Scientific, USA), respectively. Diaminobenzidine (DAB) was used for hematoxylin counterstaining.

Two pathologists analyzed blindly the prepared sections. If there were discrepancies in the samples’ description, third pathologist was invited. In heterotopic implantations, we assessed the morphological signs of the severity of dermal infiltration in the implantation area by macrophages, lymphocytes and mast cells, and also analyzed the degree of infiltration of the implants by cells and their resorption using a semi-quantitative method on a 5-point scale (Table S1, Supplementary materials).The morphometric analysis of the orthotopic implantation included evaluation of 11 morphological features (scar area; epithelium hypertrophy, dystrophy, and atrophy; density and collagen fibers’ architectonics; elastic fibers; lack of fibroblasts, cell infiltration and fibrosis in the muscle tissue, and vascularization) using a 4-point scale described previously [49, 50]. The scar’s and lamina propria’s thickness was measured using Leica Application Suite Version 4.9.0 software as described previously [49, 50]. The data were processed using GraphPad Prism version 10.00 for Windows (GraphPad Software, Inc.). To reveal differences and analyze the criteria values between groups, we applied the Kruskal–Wallis test and the post hoc Dunn’s test (p-value was set as < 0.05). The results on the scar’s and lamina propria’s thickness were evaluated as described above.

The figure is original, prepared by the authors.

## Results

### Characterization of MSC spheroids and bioequivalents

The cells isolated from the gingiva alveolar mucosa were proven to be MSC using standard criteria [8] (Figure S1, Supplementary materials).

**Fig. 2:**
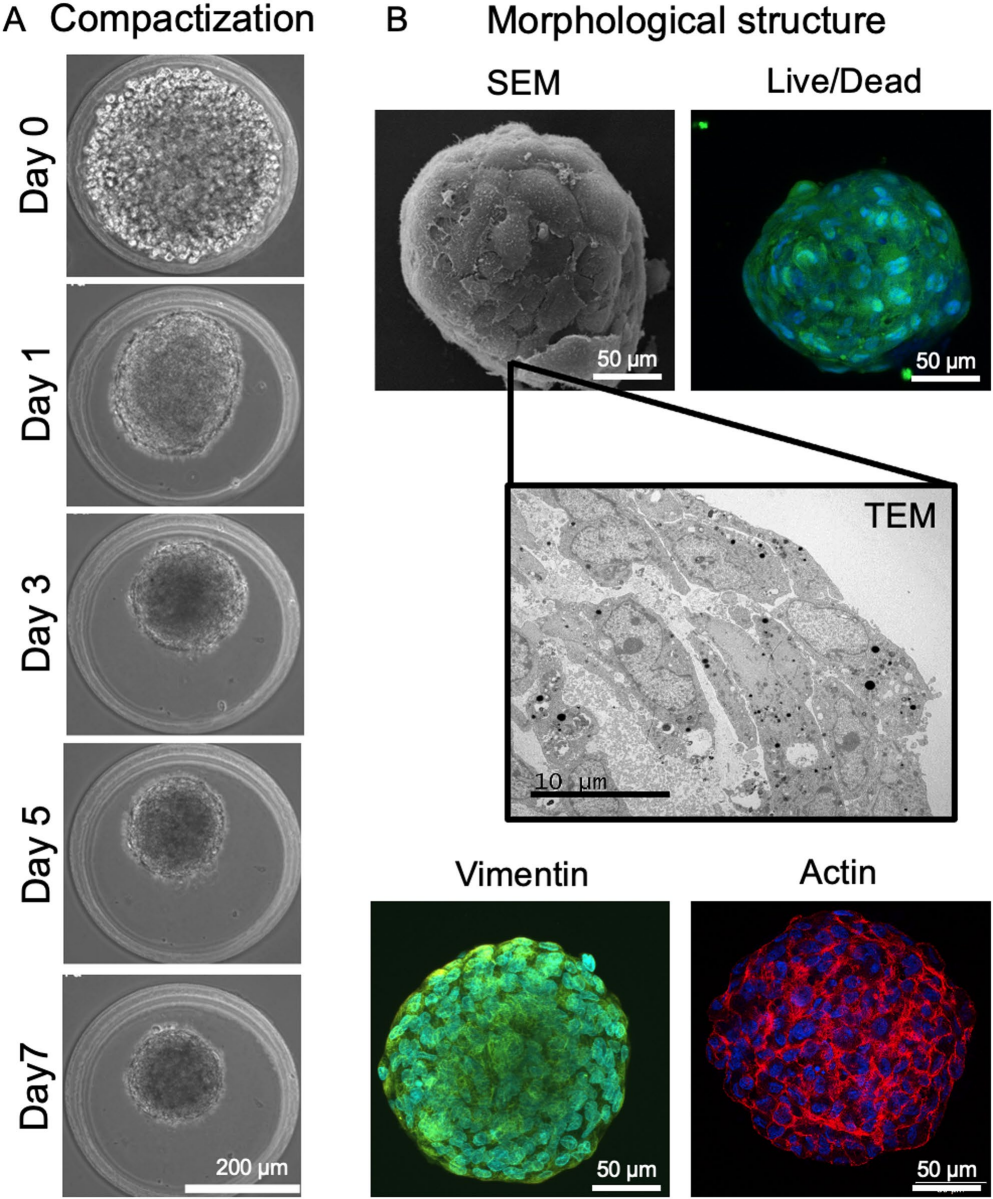
MSC spheroids’ structure: **A** – compactization dynamics (2000 cells per spheroid), phase contrast microscopy; **B** – morphological structure: scanning electron microscopy (SEM), transmission electron microscopy (TEM), laser confocal microscopy (Live/Dead; cytoskeleton markers (vimentin, actin); nuclei are counterstained with Hoechst 33258)

The spheroidogenesis was observed for 7 days. MSC aggregated on Day 1 and compactizated by Day 3 resulting in a spheroid’s diameter drop (Fig. 2). These findings are consistent with the previously reported data demonstrating a three-day-long compactization of MSC derived from the alveolar mucosa [28], and the gingiva [31] The fabricated spheroids were showed to be viable: no cells were stained by propidium iodide and most of them – by Calcein-AM (Fig. 2). The analysis of the cells located on a spheroid’s surface revealed they were densely packed, homogeneously elongated (Fig. 2), and outlined with cortical actin filaments (Fig. 2). The intermediate filament protein vimentin was expressed in the formed spheroids (Fig. 2). The ultrastructural analysis (Fig. 2) confirmed the spheroid’s zonation: at the outer zone, the densely packed cells were arranged in two or three tile-like layers; at the inner zone, they were rounded and surrounded by ECM. These findings on the formation and structure of gingival MSC spheroids are consistent with those previously reported for MSC spheroids [28, 29, 45, 56].

**Fig. 3:**
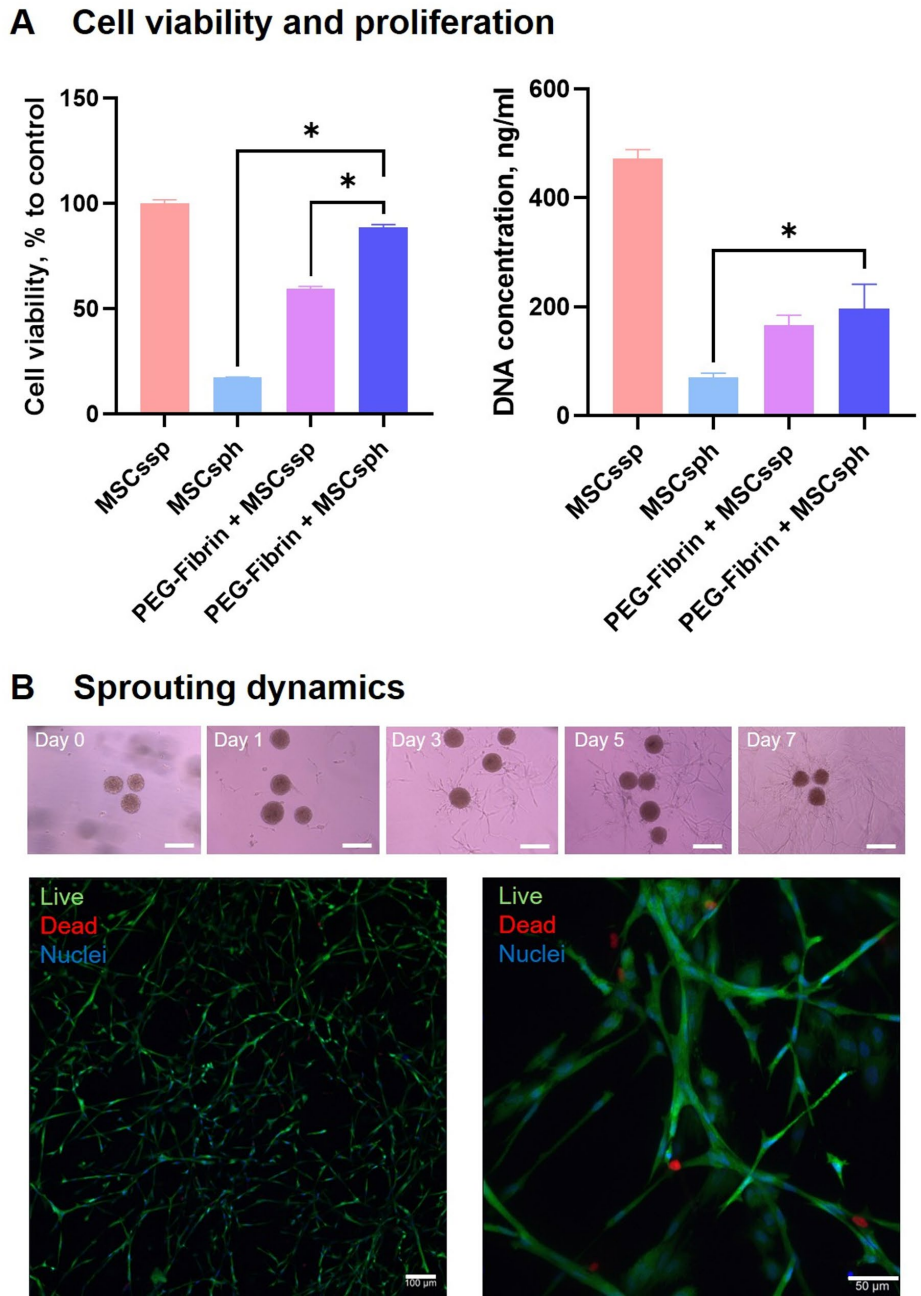
MSC bioequivalent characterization: **A** – cell viability and proliferation comparison between non-hydrogel (MSCssp and MSCsph) and PEG-Fibrin groups (*p < 0,05, 7 days of cultivation), **B** – 7-day spheroid sprouting dynamics in PEG-Fibrin (scale bar = 100 μm) and Live/Dead assay of PEG-Fibrin + MSCsph cultivated for 7 days

The used MSC cell culture was seeded as a single-cell suspension on a surface of culture plastics and cultivated for 7 days; the cell showed the high level of metabolic and proliferative activity (Fig. 3A). After being fabricated, the cells within a bioequivalent had high viability and were metabolically active; they actively migrated and proliferated (Fig. 3). Upon embedding MSCssp in the hydrogel, both metabolic activity and proliferation decreased by day 7 of cultivation. This phenomenon is commonly observed and is primarily attributed to cell adaptation to 3D conditions [6]. In contrast, the opposite trend was observed for MSCsph: by day 7, metabolic activity and proliferation were higher in the PEG-fibrin hydrogel than under non-adhesive conditions (Fig. 3A). Interestingly, in 3D conditions, both metabolic and proliferative activity were elevated in the MSCsph group. These results suggest that while MSCssp proliferate most actively in 2D conditions, spheroids function as more effective cellular units within hydrogels [7]. These conclusions are supported by visual analysis of spheroid sprouting (Fig. 3B). Cell outgrowths began to appear 24 h after seeding in the PEG-fibrin hydrogel, and by day 7, the sprouts had reached lengths of 400–500 μm. Live/dead staining of the 7-day bioequivalents revealed no dead cells, and overall cell viability remained comparable to that of non-immobilized spheroids.

### Heterotopic implantation

On Day 7, in Group 1 (PEG-Fibrin) (Figure S2 and S3, Supplementary materials), the epidermis and dermis at the implantation area were of normal structure, with only a slight increase in the content of lymphocytes, macrophages and mast cells in the dermis. The histoarchitecture of the collagen fibers in the dermis was normal. The dermis contained numerous hair follicles and glands of normal structure. The implant was represented by a fibrin mass stained light blue by Mallory’s method and retained its mostly intact fine-fibrous acellular structure. Only in the marginal areas was it slightly loosened and surrounded by an accumulation of lymphocytes, neutrophils, macrophages and fibroblasts. Inside the implant, cells and collagen fibers were virtually absent. Macrophage resorption was observed in the marginal areas of the implant.

At the same time point, in Group 2 (PEG-Fibrin+MSCsph) (Figure S2 and S3, Supplementary materials), compared to Group 1, the dermis infiltration with lymphocytes and macrophages increased, the content of mast cells remained at the same level. The histoarchitecture of collagen fibers of the dermis was unchanged. The marginal implant’s areas were infiltrated with a thick layer of cellular elements: macrophages, lymphocytes, neutrophils and fibroblasts. Large foci of loosening with cells growing into them and single collagen fibers appeared in the fine-fibrous structure of the implant. Macrophage resorption of the implant slightly increased.

On Day 21 in Group 1 (PEG-Fibrin) (Figure S2 and S3, Supplementary materials), compared to Day 7, the infiltration with lymphocytes and macrophages and the content of mast cells in the dermis did not change significantly. The fibrin implant decreased in size and loosened, and was surrounded on the outside by a wide layer of granulation tissue with occasional giant cells. Cells, mainly macrophages, migrated into the implant. The implant resorption was active, more intense than at the previous time point.

At the same time point, in Group 2 (PEG-Fibrin+MSCsph) (Figure S2 and S3, Supplementary materials), the immune cell infiltration and mast cell content slightly increased compared Group 1. Numbers of with lymphocytes, macrophages and giant multinucleated cells also increased in the peri-implant granulation tissue. The implant was significantly frayed, multiple foci of immune cells were observed in its thickness, and resorption was active.

After implantation of PEG-fibrin gels without spheroids at all time points, the increased infiltration by lymphocytes, macrophages and mast cells was observed. At the same time, cells migrated into the thickness of the implants and facilitated their resorption. The addition of spheroids had a contradictory effect, on the one hand, enhancing inflammatory infiltration, but also promoting better penetration of immune cells into the thickness of the implant and enhancing its resorption.

### Orthotopic implantation

**Fig. 4:**
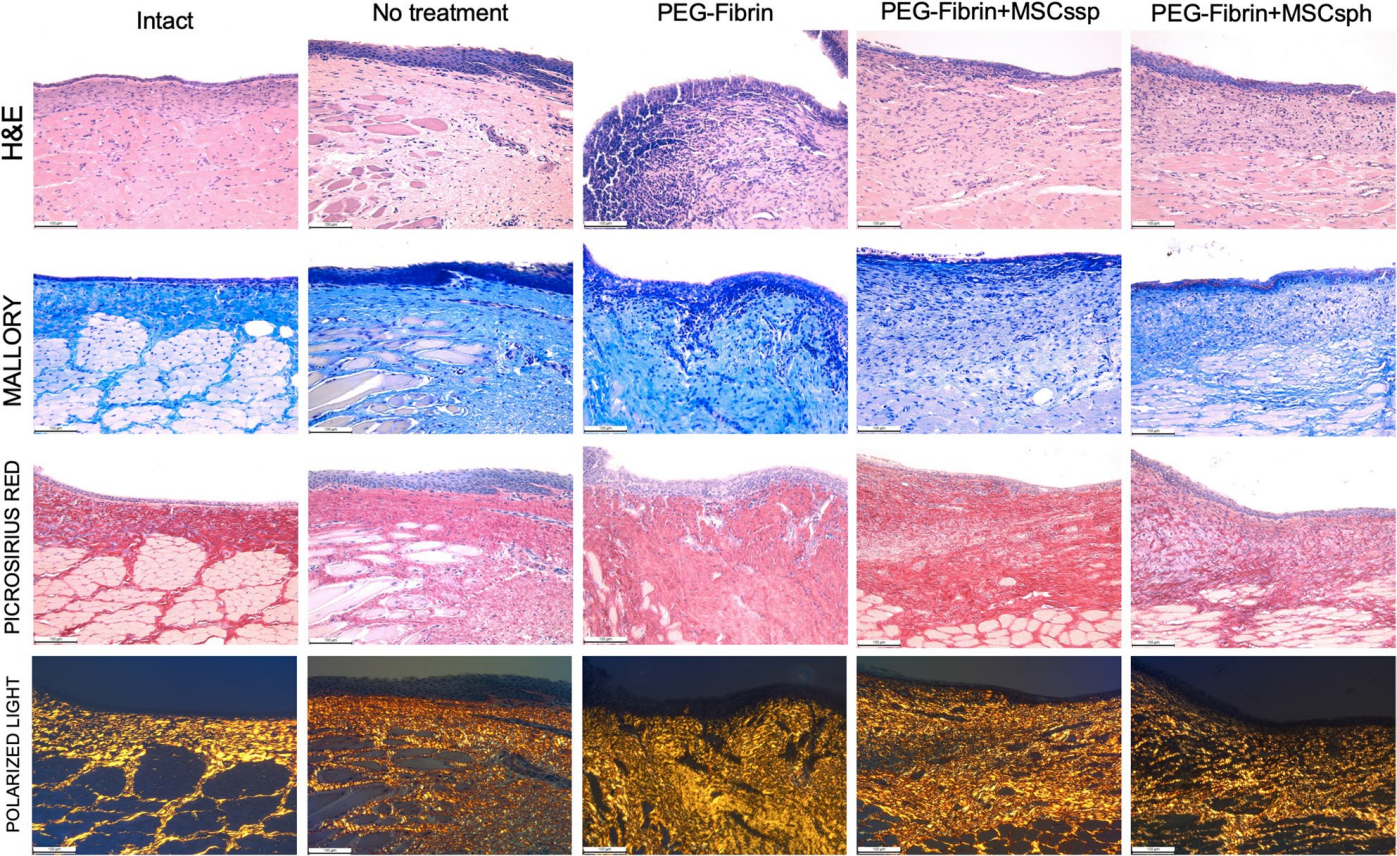
Orthotopic implantation (3 months post operation): Intact: The structure of the VF mucous membrane is normal. (H&E) The stratified squamous epithelium on the surface of the mucous, under it there is a lamina propria consisting of loose fibrous connective tissue including longitudinally oriented bundles of collagen fibers and spindle-shaped fibroblasts located between them with a small content of macrophages and lymphocytes. The deepest layer is formed by bundles of striated skeletal muscle fibers separated by thin connective tissue layers. Longitudinally arranged blue (Mallory) and red (Picrosirius red) collagen fibers gave a pronounced yellow anisotropy (polarization). Muscle fibers were white (not stained). Group 1 (No treatment): Formation of a pronounced scar in the area of the VF defect. (H&E) The hypertrophied stratified squamous epithelium. Under the epithelium, there is a scar in the lamina propria and pronounced fibrosis of the muscle fibers. Longitudinally and tightly packed blue (Mallory) and red (Picrosirius red) collagen fibers have a pronounced yellow anisotropy (polarization). Group 2 (PEG-Fibrin): The defect area is occupied by fibrous tissue with the increased immune cell infiltration. (H&E) Hypertrophied stratified squamous epithelium with signs of dystrophy. Under the epithelium, there is a dense fibrous tissue. Longitudinally and tightly packed blue (Mallory) and red (Picrosirius) collagen fibers give a pronounced yellow anisotropy (polarization). Group 3 (PEG-Fibrin+MSCssp): The defect area is occupied by dense fibrous tissue with weak immune cell infiltration. (H&E) Mature stratified squamous epithelium without signs of dystrophy. Under the epithelium, there was fibrous tissue of a dense structure with weak vascularization. Longitudinally and tightly packed blue (Mallory) and red (Picrosirius) collagen fibers have a pronounced yellow anisotropy (polarization). Group 4 (PEG-Fibrin+MSCsph): The defect area is occupied by fibrous tissue of a moderately loose structure with weak immune cell infiltration. (H&E) Mature stratified squamous epithelium of a normal structure. Under the epithelium, there is the fibrous tissue of a looser structure with weak vascularization. Longitudinally arranged blue (Mallory) and red (Picrosirius) collagen fibers give a pronounced yellow anisotropy (polarization)

In the VF mucous membrane was covered with the stratified squamous epithelium. A thin layer of the dense fibrous connective tissue was localized beneath the epithelium composing of tightly-packed collagen fibers and spindle-shaped fibroblasts with a moderate presence of macrophages, lymphocytes, and singular neutrophils. The thickness of lamia propria was 96.8 ± 28.28 μm. A fibrous structure of the layer was evident and when stained by Mallory and with PSR. The number of capillary vessels was minor. A substantial muscle layer was located adjacent to the mucous membrane with muscle bundles separated by relatively thick connective tissue membranes (Fig. 4).

On Day 3 after surgery, in Group 1 (control, no treatment) and Group 2 (PEG-Fibrin), the morphological investigation of the VF mucous membrane revealed the presence of the intensive inflammation with edema, fibrinous exudate, and a high density of neutrophils. No signs of collagen fiber synthesis were evident. Thin fibers of the remnant tissue remained embedded in deeper layers and were stained blue by Mallory and red with PSR. The muscular fiber necrosis was identified within the adjacent muscle layer. Approximately 2/3 of the defect’s central part lacked the epithelium. However, the epithelialization was observed along on the wound edges and was marked by a thin layer of proliferating keratinocytes. Epithelialization rates varied, with one instance showing over half of the defect surface epithelialized. The epithelium was growing over the edematous tissue from both edges resulting in a thin layer from one end and a relatively disorganized thicker layer from another (Figure S4A, Supplementary materials).

In Group 3 (PEG-Fibrin+MSCssp), the absence of the epithelium, pronounced edema, fibrinous exudate, and muscle tissue necrosis were observed at the center of the VF defect. The ECM at regions of the intensive inflammation lacked collagen. However, the defect’s margins included newly formed collagen fibers, which were stained blue by Mallory and red by PSR. More than a half of the defect surface was epithelialized. The epithelial layer varied in thickness and differentiation. Notably, the epithelium primarily grew over the “mature” fibrosing granulation tissue (Figure S4A, Supplementary materials).

In Group 4 (PEG-Fibrin+MSCsph), the defect surface showed the complete epithelialization. The newly formed VF mucosa section was lined with the epithelium that was thin but still thicker than in the previous study groups. This epithelium was relatively immature but displayed clear stratified squamous epithelium layers along the edges. The connective tissue beneath the epithelium contained a bulk of thin collagen fibers and singular muscle fibers that were easily detected using phase-contrast microscopy and staining by Mallory and with PSR. Significant inflammatory signs including edema, fibrinous exudate, and vast areas of muscle tissue necrosis were observed in the underlying muscle layer (Figure S4A, Supplementary materials).

**Fig. 5:**
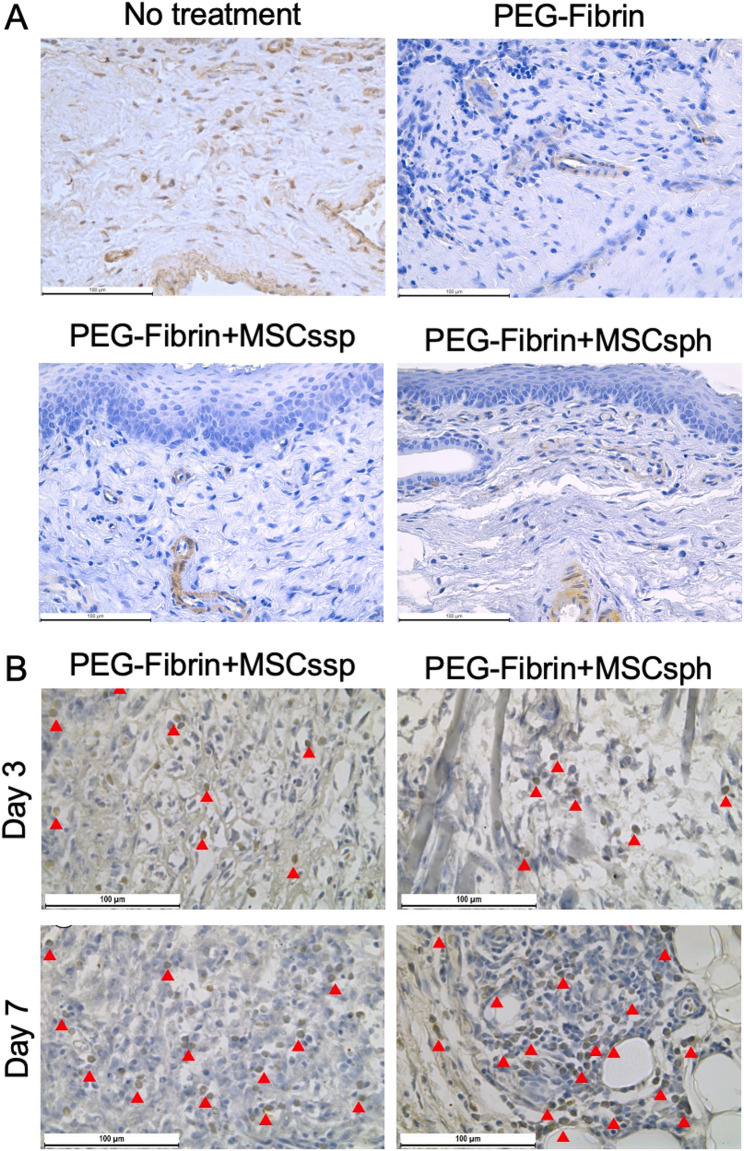
Immunohistochemical analysis of α-SMA (**A**, 3 months post operation) and nuclear human antigen (**B**, 3 and 7 days post operation) expression in the VF. **A**: No treatment – α-SMA was expressed in vascular smooth muscle cells, in numerous myofibroblasts oriented along collagen fibers and partially in epithelial cells; PEG-Fibrin – in vascular smooth muscle cells and partially in epithelial cells; PEG-Fibrin+MSCssp – in vascular smooth muscle cells, in some myofibroblasts and partially in epithelial cells; PEG-Fibrin+MSCsph – in mucosal vascular smooth muscle cells and partially in epithelial cells (scale bar = 100 μm). **B**: On Day 3, the cells with positively stained nuclei were found inside and next to the remnants of fibrin gel. The single cells were found under the stratified squamous non-keratinized epithelium, as well as between the muscle fibers near the defect site. On Day 7, the positively stained cells were found in greater numbers in the newly formed granulation wound at the defect site. They were predominantly round in shape, surrounded by fusiform fibroblasts and capillaries

Immunohistochemical analysis enabled revealing the cell presence and distribution from bioequivalents at early post-implantation stages (namely Group 3 (PEG-Fibrin+MSCssp) and Group 4 (PEG-Fibrin+MSCsph)). On Day 3, cells were observed within and adjacent to the residual gel. They were found to be singular and mainly located beneath the stratified non-keratinized squamous epithelium as well as intertwined with the muscle fibers proximal to the defect site (Fig. 5B, upper row).

On Day 7 after the secondary operation, in Group 2 (PEG-Fibrin), the VF mucous membrane defect was filled with connective tissue and was entirely epithelialized. The epithelium at the edges was nearly fully mature and resembled the normal stratified VF epithelium. However, the central area where two epithelium layers conjoined demonstrated the noticeable epithelial hyperplasia, thickening and acanthosis. The layered structure of the epithelial layer was not uniformly distinct. The granulation tissue beneath the epithelium started fibrosing while retaining a substantial number of blood vessels. An absence of edema, fibrinous exudate, and neutrophils indicated the end of the inflammatory phase. Despite this, certain regions were moderately infiltrated with lymphocytes and macrophages (Figure S4B, Supplementary materials).

In Group 3 (PEG-Fibrin+MSCssp), the defect was filled with connective tissue and covered with the mature stratified squamous epithelium with discernible layers. Some regions on the epithelium surface showed thin outgrowths of the membrane, possibly, cilia. A thin layer of fibrous connective tissue with moderate lymphocyte and macrophage infiltration located underneath the epithelium. Thin collagen fibers were distinctly visible in the newly formed mucosa area when stained by Mallory and with RSR (Figure S4B, Supplementary materials).

In Group 4 (PEG-Fibrin+MSCsph), the defect surface was fully epithelialized. The epithelium at the center of the defect demonstrated hyperplasia and thickening. The connective tissue beneath the epithelium was composed of parallel bundles of thin collagen fibers and scattered fibroblasts and macrophages. The deeper region displayed an increased number of capillaries and mature fibrous connective tissue (Figure S4B, Supplementary materials).

Immunohistochemical analysis (Group 3 (PEG-Fibrin+MSCssp) and Group 4 (PEG-Fibrin+MSCsph)) showed that by Day 7, the positively-stained cells had higher density and were predominantly located within the newly formed granulation tissue at the defect site. In Group 4 (PEG-Fibrin+MSCsph), their density was visually more prominent than that in Group 3 (PEG-Fibrin+MSCssp) (Fig. 5B, lower row). These cells were primarily round and surrounded by the fusiform fibroblasts and capillaries.

**Fig. 6:**
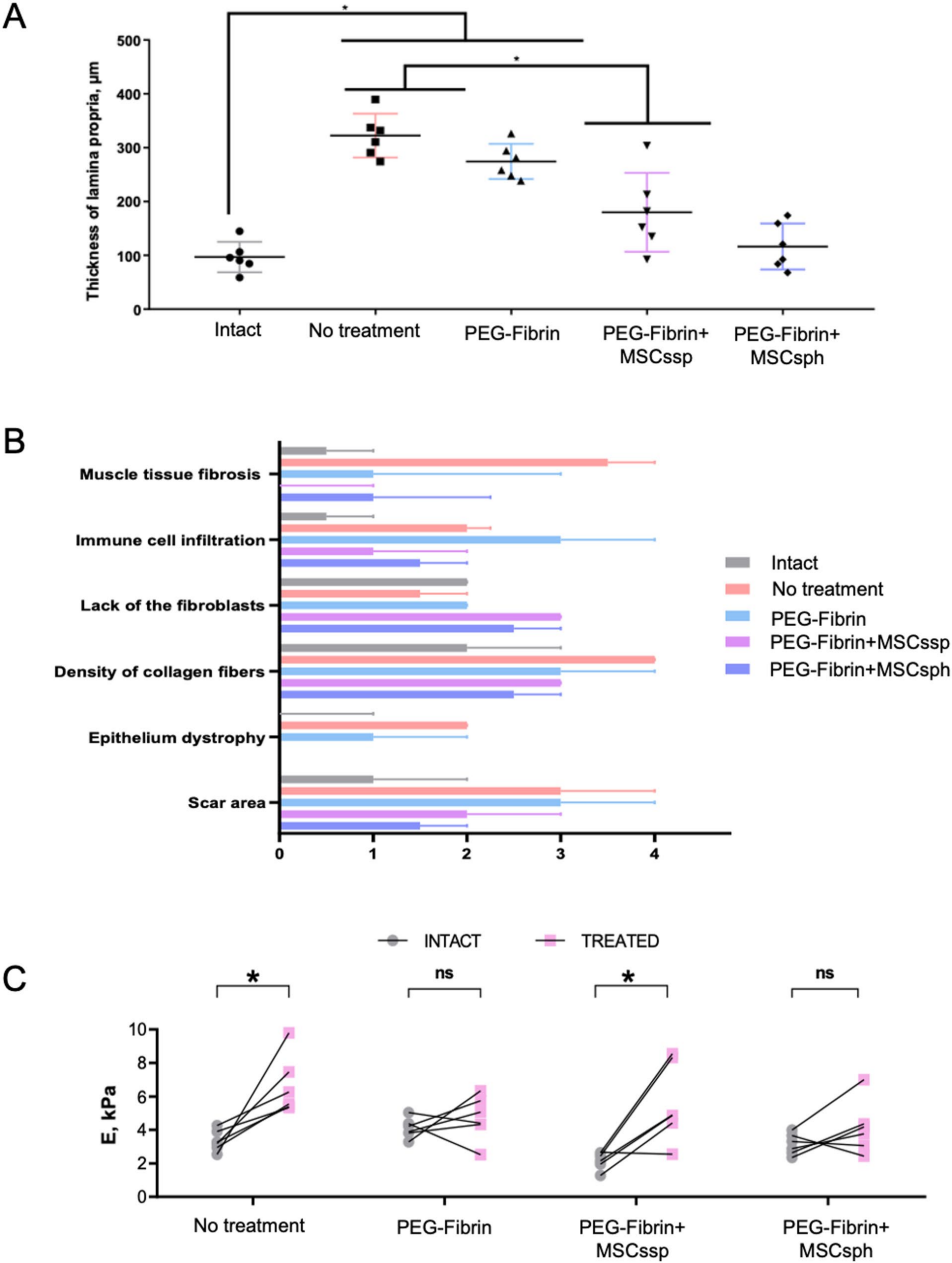
Orthotopic implantation: **A** – Morphometric analysis (median values ± interquartile range); **B** – Thickness of lamina propria (mean values ± SD, *n* = 6); **C** – Young’s modulus (E, kPa), microidentation (*n* = 6, ns: not significant, *: p-value < 0.05)

In 3 months post operation (Fig. 4), in Group 1 (control, no treatment), the VF defect area was covered with the stratified squamous epithelium with small regions of hypertrophy, atrophy, and dystrophy (Fig. 6). The lymphocyte and macrophage infiltration and vascularization were high (Fig. 4). Beneath the epithelium, the densely structured connective tissue occupied a significant area (322.45 ± 40.74 μm in thickness) compared to other groups (Fig. 4). The pronounced muscle tissue fibrosis was also observed. The α-SMA expression was noted in vascular smooth muscle cells and in a high number of myofibroblasts, which were oriented along collagen fibers (Fig. 5A, upper-left).

In Group 2 (PEG-Fibrin), the VF defects were covered with the continuous epithelium, which was predominantly stratified and squamous and had signs of dystrophy. However, some areas were covered with the ciliated columnar epithelium (Fig. 4). This indicates the lack of antifibrosis effect. The collagen fibers stained well by Mallory and with PSR exhibiting strong anisotropy in the polarized light. The collagen fibers were densely packed similar to scars in Group 1 (control, no treatment). The mucous membrane was significantly infiltrated by lymphocytes and macrophages and contained full-blooded vessels (dilated capillaries and venules) (Fig. 4). The connective tissue beneath the epithelium was relatively thick (274.42 ± 32.75 μm) and moderately loose composed of the longitudinally oriented collagen fibers and intervening fibroblasts. The thickness of lamia propria was higher than that in Group 1 (control, no treatment) (*p*<0.0001) and did not significantly differ (Fig. 4). The α-SMA expression was observed in vascular smooth muscle cells and partly in cells of the epithelial layer, no accumulations of myofibroblasts were found (Fig. 5A, upper-right).

In Group 3 (PEG-Fibrin+MSCssp), the newly formed area of the mucous membrane was covered with the mature stratified squamous epithelium with clearly distinguishable layers (Fig. 4). The number of fibroblasts was lower than that in Group 1 (control, no treatment) (*p* = 0.036). However, it was significantly lower than that in Group 1 (control, no treatment) indicating that application of MSC encapsulated in PEG-fibrin gel gave a limited therapeutic effect. This group had the least notable fibrotic changes in muscle layer compared to the intact VF (*p* = 0.002) (Fig. 6). The mature connective tissue (179.84 ± 73.34 μm in thickness) was located beneath the epithelium without dystrophic changes (*p* = 0.018 compared to Group 1 (control, no treatment)) and had a relatively loose ECM (*p* = 0.286 compared to Group 1 (control, no treatment)) and minor lymphocyte and macrophage infiltration. The thickness of lamia propria was higher than that in the intact VF (*p* = 0.0347) (Fig. 4). The α-SMA expression was revealed in vascular smooth muscle cells, in individual myofibroblasts, and partially in epithelial cells (Fig. 5A, lower-left).

In Group 4 (PEG-Fibrin+MSCsph), the newly formed mucous membrane was covered with the mature stratified squamous epithelium without signs of dystrophy (Figs. 4 and 6). The loose connective tissue consisted of longitudinally oriented collagen fibers, spindle-shaped fibroblasts, and slight lymphocyte and macrophage infiltration (Fig. 4). There were trends in decrease of collagen fiber density (*p* = 0.09), number of fibroblasts (*p* = 0.127), and muscle tissue fibrosis (*p* = 0.102) compared to the intact VF. In most samples, the lamia propria was relatively thin (116.42 ± 42.77 μm) and loose indicating the absence of fibrosis. It was the only group where the thickness of lamia propria did not differ from the intact VF (*p* = 0.947). The thickness was also significantly lower than that in Group 1 (control, no treatment) (*p* < 0.001) (Fig. 4). These results show the application of the bioequivalent based on MSC spheroids is superior to that performed using one based cell suspension. The α-SMA expression was observed in smooth muscle cells of vessels of different sizes similar to the normal VF and partially in epithelial cells (Fig. 5A, lower-right).

### Analysis of mechanical and vibration properties

We measured the Young’s moduli of the intact and treated or non-treated VF, and their values were compared pairwise in each group. In Group 1 (control, no treatment), the Young’s modulus of the scar was found to be 6.63 ± 1.75 kPa that was significantly higher (in 1.9 times) than that of the intact tissues (3.35 ± 0.63 kPa; *p* ≤ 0.05) (Fig. 6C). In Group 2 (PEG-Fibrin), no significant difference was revealed between the intact and treated VF (4.08 ± 0.66 vs. 4.73 ± 1.33 kPa, respectively) (Fig. 6C). In Group 3 (PEG-Fibrin+MSCssp), interestingly, the treated VF had significantly higher the Young’s modulus that the native tissue (5.60 ± 2,63 vs. 2.23 ± 0.57 kPa, respectively; *p* ≤ 0.05) (Fig. 6C). Furthermore, this group demonstrated the greatest variability in Young’s modulus among all groups, which may reflect heterogeneity of regeneration or uneven tissue remodeling. In Group 4 (PEG-Fibrin+MSCsph), the values of the Young’s modulus of both intact and treated VF were similar and no significant difference was revealed (3.19 ± 0.69 vs. 4.13 ± 1.58 kPa, respectively) (Fig. 6C).

The vibration properties (Table 1) were characterized using three main vibration frequency ranges able to be registered – f1, f2, and f3, where f1 is the lowest range and f3 – the highest one. To excite vibrations, we varied the air flow pressure; a variable pressure air stream was used to excite VF vibrations. It was found that when the airflow was between 50 mm Hg and 60 mm Hg, the VF produced a white vibration noise between 100 Hz and 10 kHz.

The Shapiro-Wilk test showed no significant deviation from normality (*p* = 0.849). Pairwise comparison of the groups in these areas (ANOVA analysis of variance) showed no significant differences between the experimental groups and the intact VF. No differences were found in the fundamental frequencies of excited vibrations between the intact VF and the groups with implantation of the fibrin hydrogel-based material (Table 1). The frequencies of the excited modes of the treated VF were comparable to those of the intact VF.

**Table 1:** Vibration frequency ranges f1, f2, f3 (Hz) (mean values ± SD)

	Intact	Treated
PEG-Fibrin		
f1	269.8 ± 68.48	303.8 ± 98.71
f2	544.2 ± 145.57	483 ± 79.73
f3	795 ± 237.11	681.33 ± 137.41
PEG-Fibrin+MSCssp		
f1	301 ± 35.45	273.66 ± 52
f2	602.2 ± 71.33	543.67 ± 111.89
f3	908.8 ± 116.65	823.33 ± 175.30
PEG-Fibrin+MSCsph		
f1	274.2 ± 62	277.4 ± 44.43
f2	545.2 ± 125.84	560.8 ± 87.80
f3	843.25 ± 200.64	834.2 ± 130.28

## Discussion

The main mechanism of MSC pro-regenerative properties in the VF is caused by their paracrine effects. Compared to one derived from a monolayer culture, the secretome from spheroids has shown the enhanced activity due to both morphological and molecular changes related to cell interactions [13, 18, 26, 36]. These changes include the increased synthesis of agents associated with the stress response to the cellular re-organization and metabolic reconfiguration, and the formation of an MSC’ niche in spheroids [5]. Specifically, spheroids from gingival MSC exhibited the increased expression levels of stemness regulators such as Stro-1, CXCR-4, OCT-4, and Nanog 2, and demonstrated high resistance to apoptosis and oxidative stress compared to monolayer cultures. Such spheroids were shown to have high efficacy in reversing body weight loss and promoting the regeneration of the disrupted epithelial lining of the mucositic tongues [31, 57]. The secretome of MSC’ spheroids has also been revealed to significantly affect the polarization of macrophages into an anti-inflammatory M1 phenotype and reduce the level of pro-inflammatory M2 macrophages [3, 43]. A study has shown that hydrogel implementation can enhance cellular secretory output, particularly in low-stiffness hydrogels such as fibrin [9, 15, 32]. Together, employing spheroids rather than suspensions would provide higher yields of secreted factors both by increased cell density and secreting activity. Additionally, hydrogels can serve as a protective medium for vesicles secreted by MSCs by providing high water content and cell/vesicle membrane protection [15]. Using the hydrogels as a carrier for vesicle-containing conditioned medium is an effective strategy for wound healing applications [39].

The effects of MSC implantation observed by us in the experiment groups were appeared as pronounced thin collagen fibers located linearly that is a clear consequence of the MSC features mentioned above and ECM stabilization. Compared to other groups, the full epithelization of the VF’ mucous membrane was noticed by Day 3 when MSC spheroids were used; its histological structure was similar to that of the native tissues. The same results were observed by Na et al., [38]. They showed that the use of MSC at a defect side enabled the restoration of collagen fibrils’ microarchitectonics; and while MSC spheroids applied, the fibers were more dense and well oriented. Choudhury et al., also demonstrated that compared to single cell suspension, MSC spheroids significantly induced re-epithelization and caused an insignificant scar at a macro- and micro-level [11].

Moreover, the implantation of cells as spheroids increases their survivability and ensures the prolongation of their presence at a defect site [2, 21, 25, 54, 55]. This becomes a significant benefit while treating the VF because of their low accessibility and low manipulation tolerance by patients. We showed that compared to a single cell suspension, MSC spheroids remained at the implantation site for at least 7 days. These data correlate with the previously reported one by Regmi et al. [44].

One of the parameters of the successful VF’ restoration is their mechanical lability and vibration ability ensured by anatomical structure. Our study revealed that when MSC spheroids implanted, the lamina propria was relatively thin (116.42 ± 42.77 μm) compared to that in the control (322.45 ± 40.74 μm; р<0.0001) and treated with MSC suspension (179.84 ± 73.34 μm; р<0.0001). Similar positive effects were reported by Lee et al. [30] who injected spheroids from adipose tissue-derived MSC into old rats as a model of presbylaryngis (the rima glottidis’ area was significantly reduced, and the difference in VF’ phases during phonation was statistically insignificant). The analysis of mechanical properties revealed that the Young’s modulus values comparable to those of the intact tissues was common for the VF treated with MSC spheroids immobilized in PEG-fibrin hydrogel system and only PEG-fibrin; interestingly, the use of MSC suspension was less effective than that of only gel. The changes in Young’s modulus values at any range direction reflect the scar formation appearing as rougher or looser tissue at a defect side that cannot be considered positive for the restored VF.

## Conclusions

The achieved results showed that both types of a bioequivalent (suspension- and spheroid-based) lead to the pronounced VF restoration after scar excision. Nevertheless, the use of MSC spheroids ensured their better retaining at a defect site that caused more efficient regeneration, and the histological structure and mechanical properties of the treated VF was more similar to those of the intact VF.

## Electronic Supplementary Material

Below is the link to the electronic supplementary material.

Additional file 1.

## Data Availability

All data generated or analyzed during this study are included in this published article.

## Abbreviations

α-SMA: α-Smooth muscle actin
DAB: Diaminobenzidine
ECM: Extracellular matrix
MSC: Mesenchymal stromal cells
PBS: Phosphate-buffered saline
PSR: Picrosirius red
SD: Standard deviation
