# Supplementary material for: SPHERpower: MSC spheroid-based bioequivalent lead to the efficient restoration of the scarred vocal folds
Source: Stem Cell Res Ther. 2026 May 30;17:267. doi: 10.1186/s13287-026-05067-5 (PMC13439855; doi:10.1186/s13287-026-05067-5)
Supplement: Supplementary file 1 [file 13287_2026_5067_MOESM1_ESM.docx]

**Supplementary materials**

Figure S1. Characterization of MSC cell culture: A – monolayer culture, Passage 3 (phase contrast microscopy); B – cell differentiation (chondro- (Alcian Blue), adipo- (Red Oil O), and osteogenic (Alizarin Red S) lineages); C – immunophenotyping, Passage 3 (most cells expressed CD29, CD44, CD73, CD90, and CD105). The cells had typical spindle-shaped morphology and showed the ability to adhere to a surface, to migrate and to proliferate resulting in the monolayer formation as well as to differentiate into three lineages (chondro-, adipo-, and osteogenic ones) (Figure S1). Immunophenotyping revealed that the negative markers’ (CD19, CD11b, CD34, CD45, and HLA-DR) expression was typical for less than 3%, and the majority of the cells isolated (higher than 99%) expressed CD29, CD90, CD44, CD73, and CD105.

Figure S2. Heterotopic implantation (Day 7 and 21 after implantation), hematoxylin and eosin (H&E), Picrosirius red and Mallory’s stainings, magnifications ×50 and ×200:

Group 1 (PEG-Fibrin): Day 7. General view of the skin (epidermis, dermis, hypodermis) and the implant in the hypodermis. Homogeneous fine-fibrous structure of the implant with areas of loosening, numerous immune cells on the surface of the implant, growth of individual cells into the thickness of the implant. Collagen fibers of the dermis and hypodermis were stained red. The fibrin base of the implant was stained blue. Day 21. The area of reaction to the implant expressed moderate signs of inflammation. Foci of macrophage growth into the thickness of the implant. Dermis in the area of implantation with weak infiltration with lymphocytes and macrophages. The fibrin base of the gel implant without spheroids was stained blue.

Group 2 (PEG-Fibrin+MSCsph): Day 7. Large areas of loosening in the fine-fibrous structure of the implant, numerous immune cells on the surface of the implant, their active growth into the thickness of the implant. Scarce collagen fibers in the immediate vicinity of the implant were stained red. The fibrin base of the implant, inside which immune cells were determined, was stained blue. Day 21. The implantation site was characterized by a thick capsule and cell growth into the gel. Foci of immune cell growth into the implant. Collagen fibers of the peri-implant granulation tissue were stained red. The fibrin base of the implant was stained blue, immune cells were determined in the implant.

Figure S3. Heterotopic implantation: morphometrical analysis of tissue reaction. Median values with interquartile range (* - p ≤ .05, ** - p ≤ .01, *** - p ≤ .001, **** - p ≤ .0001).

Figure S4. Orthotopic implantation (3 (A) and 7 (B) days post operation).

Table S1. Criteria for semi-quantitative assessment of morphological parameters of skin samples in the heterotopic implantation area on a 5-point scale.

| Morphological parameter | Criteria | Scores |
| --- | --- | --- |
| Dermal infiltration with lymphocytes and macrophages | Absent | 0 |
|  | Singular immune cells (1-10 per field of view at 200x) | 1 |
|  | Several foci of immune cells (10-30 per field of view at 200x) | 2 |
|  | Multiple foci of immune cells (30-50 per field of view at 200x) | 3 |
|  | Severe diffuse infiltration with immune cells (more than 50 per field of view at 200x) | 4 |
| Mast cells in derma | Absent | 0 |
|  | Singular cells (1-2 per field of view at 200x) | 1 |
|  | Sparse small foci of cells (3-5 per field of view at 200x) | 2 |
|  | Sparse large foci of cells (5-10 per field of view at 200x) | 3 |
|  | Multiple large foci of cells (more than 10 per field of view at 200x) | 4 |
| Immune infiltration of the implant | Absent | 0 |
|  | Singular immune cells on the implant surface, there are no areas of implant loosening | 1 |
|  | A layer of immune cells on the implant surface, individual small foci of immune cells in the thickness of the implant, small areas of loosening of the implant are found | 2 |
|  | Multiple foci of immune cells within the implant, multiple areas of implant loosening | 3 |
|  | The entire implant is permeated with numerous immune cells and is significantly loosened | 4 |
| Implant resorption | Absent | 0 |
|  | Resorption observed only in marginal areas of the implant | 1 |
|  | Resorption observed along the entire periphery of the implant, but absent in the thickness | 2 |
|  | Resorption observed along the entire periphery of the implant and focal resorption in the thickness of the implant | 3 |
|  | The entire implant is actively resorbed with the resorbed areas being replaced by connective tissue | 4 |
